# Pharmacotherapeutic Options for Visceral Leishmaniasis—Current Scenario

**Published:** 2009-01-23

**Authors:** Krishna Pandey, Prabhat Kumar Sinha, Vidyanand Ravi Das, Sanjiva Bimal, Shubhankar K. Singh, Pradeep Das.

**Affiliations:** Rajendra Memorial Research Institute of Medical Sciences (ICMR), Agamkuan, Patna-800 007, Bihar, India

**Keywords:** visceral leishmaniasis, sodium stibogluconate, Amphotericin B, miltefosine, paromomycin, sitamaquine

## Abstract

Visceral leishmaniasis (VL) or Kala-azar is a protozoal disease, which was previously regarded as one of the most neglected tropical diseases. Management of this disease is quite difficult, because it is said to affect the poorest of the poor. Previously Sodium Stibogluconate (SSG) was regarded as the gold standard treatment for VL. But due to the increasing unresponsiveness, to this drug various other drugs were tried and are still being tried. Pentamidine is very toxic and has been discarded of late. Amphotericin B and its lipid formulations are very effective but require hospital admission and monitoring. Oral drugs like Miltefosine have already been launched. An amino glycoside Paromomycin and another oral drug Sitamaquine are in the pipe line. Interferon gamma has been used with discouraging results.

Visceral leishmaniasis (VL) or Kala-azar is vector borne disease, caused by a protozoan parasite, *Leishmania donovani* and transmitted by the bite of female sand-fly, *Phlebotomus argentipes* in India. It can also be caused by *L. infantum*, *L. chagasi*, and viscerotropic strains of *L. amazonensis*, *L. maxicana* and *L. tropica*. Moreover, there are other vectors than *P. argentipes*. It is prevalent in more than 80 countries in Asia, Africa, Southern Europe and South America. It is estimated that every year about 5 00,000 new cases occur worldwide. Of these, 90% cases occur in five countries namely India, Nepal, Bangladesh, Brazil and Sudan. India alone contributes to 50% of the global burden of VL yearly and 90% of this occur in North Bihar only.^1^ Epidemics have occurred in India more so in the state of Bihar. The disease is clinically characterized by prolonged fever, hepatosplenomegaly and pancytopenia. These days, there are challenges in the chemotherapy of kala-azar like (a) widespread resistance to antimonials, (b) safe and cost effective medications with less toxicity, (c) HIV leishmania co-infection and its rising incidence and (d) the non availability of an effective vaccine.^2^

## Parenteral Drugs

### Sodium stibogluconate

It was the initial drug to be used for the management of VL, in the dose of 10 mg/Kg for 6 to 10 days. But due to increasing unresponsiveness, its dose was modified and the standard WHO recommendation, at present, is 20 mg/Kg for 30 days deep intra muscular. In one study, the unresponsiveness to sodium stibogluconate (SSG) in Bihar State and particularly in highly endemic districts was of the order of 60%. SSG still remains the drug of choice for VL outside Bihar.^3^

The exact mechanism of action is still not very clear. It is said to influence the bioenergetics of leishmania parasite by inhibiting parasite glycolysis, fatty acid ß oxidation and inhibition of ADP phosphorylation. It may also promote efflux of thiols mainly glutathione and trypanothione.^4^ The main toxic effects are cardiotoxicity, especially myocarditis and arthritis besides acute tubular necrosis in a few cases. The cure rate at present has come down to as low as 45% in the endemic districts of Bihar.

### Pentamidine Isethionate

It is an aromatic diamidine and was previously, used as a second line drug. Its anti-leishmanial activity, is due possibly to polyamine biosynthesis and its effect on mitochondrial membrane potential. The dose is 4 mg/Kg intravenously slowly on alternate days for 15 injections and has a present cure rate of about 70%. However, because of multiple toxic effects like anaphylactic shock, nephrotoxicity and diabetes mellitus (in about 10% of the patients) its use has been restricted and has been discarded of late.

### Amphotericin B

This is an anti-fungal macrolide antibiotic, which was shown to have anti-leishmanial activity in the late 1960s. Its main mechanism of action, is due to its selective affinity for position-24 substituted sterols mainly ergosterol. It also causes formation of aqueous pores resulting in cell lysis. At a dose of 1 mg/Kg body weight for 15 injections in 5% dextrose intravenously, either on alternate days or daily the cure rate has been shown to be as high as 98%.^5^ Primary unresponsiveness and relapses are very uncommon. The main side effects include chills and rigor, thrombophlebitis, occasionally myocarditis, hypokalaemia, renal dysfunction and death. The drug needs prolonged hospitalization and close monitoring for toxicity including electrocardiogram (ECG) and serum electrolytes.

Lipid formulations of Amphotericin B have advantages like (a) minimum toxicity, (b) short duration of therapy and (c) target specificity (i.e. it is delivered directly to the parasitized reticuloendothelial system cells). The main draw back is, however, its high cost which limits its use in developing countries like India. Three lipid formulations are available (a) liposomal Amphotericin B (Ambisome), (b) Amphotericin B lipid complex (Abelcet) and (c) Amphotericin B colloidal dispersion (Amphocil). In Indian patients, Ambisome in a dose of 3 to 5 mg/kg for five days or 15 mg/kg as a single dose cured all patients (100%) even in refractory cases.^6^ The cost of a single dose of 5 mg/kg of Ambisome for a 30 kg patient is about Rs. 27,000 (US$574.47) as compared to Rs. 2700 (US$57.45) for a typical course of Amphotericin B deoxycholate. All in all, Amphotericin B and its lipid formulations are probably the best in the list of drugs used for treating VL at present.

### Paromomycin

It is an injectable aminoglycoside anti-leishmanial drug.^7^ In Phase III multicentric trial (of which Rajendra Memorial Research Institute of Medical Sciences, Patna was also a part), a final cure rate of 94% was reported. The drug was used in the dose of 15 mg/kg/day for 21 days intramuscularly. The main side effects, as with other aminoglycosides, nephrotoxicity and ototoxicity fortunately were of a very low degree. This drug has the potential to replace SSG as the first line of drug for VL. Problem of treatment failure and relapses have to be assessed when used as monotherapy. Phase IV trials of the drug are due to start soon at various clinical centres of Bihar.

## Oral Compounds

### Miltefosine

This is an alkyl phospholipid compound which was previously used as a topical application for the skin metastasis of breast cancer. The main anti-leishmanial activity is due to modulation of cell surface receptors, inositol metabolism, phospholipase activation and protein kinase C besides mitogenic pathways resulting in apoptosis.^8^ This drug has a long half life of about 150 to 200 hours, thereby raising concerns for emergence of resistance. 9 The dose is 2.5 mg/kg/day in divided doses orally for 28 days. Phase III and Phase IV trials at this center and various other centers have demonstrated a final cure rate of about 94%. It is one of the few drugs which even though being an anti-neoplastic agent, stimulate the bone marrow thereby leading to correction of pancytopenia. The main side effects of the drug include gastrointestinal disturbances like nausea, diarrhoea and vomiting in about 20 to 30% of the cases. The symptoms are transient or reversible despite (during) ongoing treatment and not a cause of major concern. In the phase III trial, alanine transaminase (ALT) and aspartate transaminase (AST) levels were raised in the first week (AST) or occasionally towards the end (both AST and ALT). These relations did not require treatment modification. Renal toxicity was observed in a few cases. The drug is not to be used in pregnancy due to its possible teratogenic effect. However, in phase IV trial, three female patients became pregnant during the follow up period. All the three had full term normal pregnancy and the fetuses were devoid of any teratogenic effects. This drug can prove to be a first line drug and the expert committee has recommended for its use in the elimination of VL programme in India.

### Azoles

These are sterol biosynthesis inhibitors and were shown to be effective against wide range of promastigotes and amastigotes. But their antileishmanial activity was not enough to induce clinical cure by themselves. Ketoconazole and fluconazole are two drugs that were used for VL in this group.

### Sitamaquine

This is an orally active 8-aminoquinoline analogue and its effectiveness in VL was validated in animal models. It is still in phase I and II trial stages. In one of the multicentric phase II trials (of which this center is also a part) it is being used in the dose of 2 mg/kg/day orally for 21 days. In another previous phase II trial in Kenya as well as India, the cure rate was about 50%. The main toxic effects were renal, like glomerulonephritis and acute tubular necrosis.^10^

### New investigational products in pre-clinical phases

Licochalcone A, derived from Chinese liquorice plant *Glycyrrhiza*, has shown reasonable oral efficacy in experimental models of VL as well as Cutaneous leishmaniasis (CL). One derivative gave 97% suppression of *L. donovani* liver amastigotes in a hamster model. Another compound from a Vietnamese plant *Maesa balanasae* showed significant activity in VL models. Isopropylquinolines, isolated from *Galipea longiflora inboligia*, has also shown activity in VL models. Biphosphonates, used for osteoporosis, have been found to be effective against both VL and CL. Two of these drugs, risedronate and pamidronate, have also been found to be effective.^11^

### Immunomodulators

Due to the intracellular nature of leishmania parasite, VL patients usually require an interferon-gamma (IFN-γ) dominant T-helper cell type 1 (Th1) pro-inflammatory response for clearing of the parasite. As such, it was felt that the IFN-γdominant inflammatory mechanism, would be required to support the efficacy of conventional anti-leishmanial chemotherapy.^12^ However, clinical trials with gamma interferon in combination with SSG, were very disappointing in that they gave a cure rate of about 50%. Besides, this is very costly and requires cold chain for storage.

### HIV–leishmania co-infection

This combination is assuming dangerous proportions worldwide, more so in Southwest Europe and India. The management of such cases is very difficult because of (a) frequent relapses, (b) dosage and duration of the drugs to be administered have not been validated and (c) drug-drug interactions.

Pentavalent antimonials need to be given for a prolonged period which can lead to severe cardiac toxicity. The cure rate is low, about 50 to 60%. Amphotericin B in the dose of 1 mg/kg/day for 28 days had a cure rate of about 62%. Amphotericin B lipid complex in the dose of 4 mg/kg/day on days 1–5, 10, 17, 24, 31 and 38 was well tolerated. Miltefosine was used in one patient with good results.^13^ This inspired a whole series of compassionate use in patients over the following years.^14^,^15^ These days various drug combinations are being tried. These include (a) SSG 20 mg/kg/day for 28 days + Paromomycin 50 mg/kg/day for 28 days,^16^ (b) SSG 20 mg/kg/day for 28 days + Amphotericin B 1 mg/kg/day for 28 days, (c) SSG 20 mg/kg/day for 28 days + Allopurinol 20 mg/kg/day for 28 days,^17^ (d) SSG 20 mg/kg/day for 28 days + gamma interferon 100 μg/m^2^/day for 28 days,^18^ (e) Miltefosine 2.5 mg/kg/day for 28 days + SSG 20 mg/kg/day for 28 days or Amphotericin B 1 mg/kg/day for 28 days and (f) Miltefosine 100 mg/day for 28 days + Amphotericin B 1 mg/kg/day for 28 days. These drugs are being tried at various centres all over the world. All these patients, are also to be administered highly active anti-retroviral therapy (HAART) depending on CD_4_ count level (if less than 200/μl), besides treatment of other opportunistic infections. However, drug interactions with HAART, mainly non-nucleoside reverse transcriptase inhibitors like nevirapine, protease inhibitors like indinavir, sequanavir etc. and anti-tuberculous drugs like rifampicin need to be taken into consideration, as most of these patients suffer from tuberculosis as well.^19^

In conclusion, it is to be stated that in the last three decades unresponsiveness to pentavalent antimonials in certain parts of Bihar has arisen to >60%. As a result, Amphotericin B is considered to be the best drug at present. However, it is relatively much costlier as compared to SSG. Newer drugs like Miltefosine are very promising, but costly. Paromomycin seems to be another effective therapeutic option with low cost. Future strategies should be made to save new potent drugs from acquiring resistance and better management of co-infection cases. This can open up a gate for combination therapy. A few new indigenous plant extracts as well as synthetic compounds, having anti-leishmanial activity, need to be explored for its safety and efficacy in VL treatment through clinical trials. Further more, studies should be under taken to test the efficacy of second generation anti-leishmanial vaccine as prophylaxis.
